# 2-Hy­droxy-*N*′-methyl-5-nitro­benzohydrazide

**DOI:** 10.1107/S1600536813019776

**Published:** 2013-07-24

**Authors:** Ming Yang, Chengzhi Jin, Xiaodong Zhou, Longfei Jin

**Affiliations:** a College of Chemistry and Material Science, South-Central University for Nationalities, Wuhan 430074, People’s Republic of China

## Abstract

In the title compound, C_8_H_9_N_3_O_4_, there are two mol­ecules in the asymmetric unit, one of which is in the zwitterionic form. The zwitterion contains an intra­molecular N—H⋯O hydrogen bond and the other mol­ecule contains both an intra­molecular N—H⋯O and an intra­molecular O—H⋯O hydrogen bond. In the crystal, N—H⋯O and N—H⋯N hydrogen bonds link the mol­ecules, formimg a two-dimensional network parallel to (10-1).

## Related literature

For the biological activities of salicylhydrazide derivatives, see: Bagchi *et al.* (2004 ▶); Thompson *et al.* (2004 ▶); Al-Mawsawi *et al.* (2007 ▶). For metal complexes involving derivatives of the title compound, see: Jin *et al.* (2006*a* ▶,*b* ▶). For related crystal structures, see: Liu *et al.* (2006 ▶); Luo *et al.* (2007 ▶); Xu & Liu (2006 ▶); Zhang (2012 ▶).
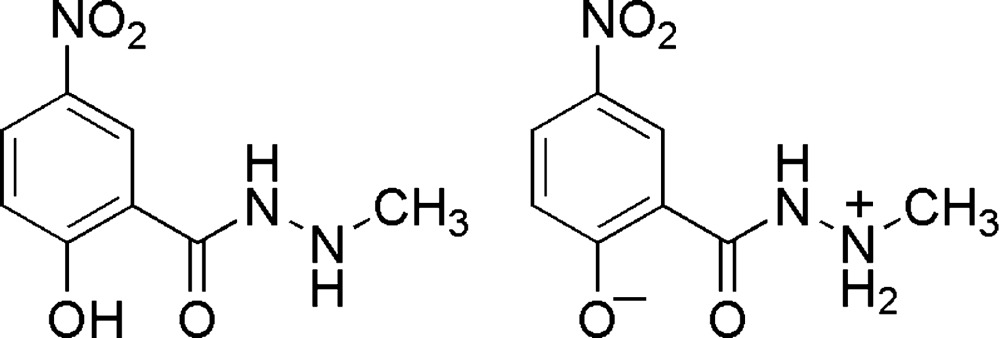


## Experimental

### 

#### Crystal data

C_8_H_9_N_3_O_4_
*M* *_r_* = 211.18
Monoclinic, 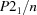

*a* = 7.3818 (15) Å
*b* = 13.106 (3) Å
*c* = 18.719 (4) Å
β = 95.25 (3)°
*V* = 1803.4 (6) Å^3^
*Z* = 8
Mo *K*α radiation
μ = 0.13 mm^−1^
*T* = 293 K
0.26 × 0.20 × 0.10 mm

#### Data collection

Bruker SMART CCD diffractometer
Absorption correction: multi-scan (*SADABS*; Sheldrick, 1996 ▶) *T*_min_ = 0.968, *T*_max_ = 0.987
11293 measured reflections
3536 independent reflections
2356 reflections with *I* > 2σ(*I*)
*R*_int_ = 0.044

#### Refinement

*R*[*F* ^2^ > 2σ(*F* ^2^)] = 0.058
*wR*(*F* ^2^) = 0.142
*S* = 1.06
3536 reflections
291 parameters
5 restraints
H atoms treated by a mixture of independent and constrained refinement
Δρ_max_ = 0.22 e Å^−3^
Δρ_min_ = −0.18 e Å^−3^

### 

Data collection: *SMART* (Bruker, 2001 ▶); cell refinement: *SAINT* (Bruker, 2001 ▶); data reduction: *SAINT*; program(s) used to solve structure: *SHELXS97* (Sheldrick, 2008 ▶); program(s) used to refine structure: *SHELXL97* (Sheldrick, 2008 ▶); molecular graphics: *SHELXTL* (Sheldrick, 2008 ▶); software used to prepare material for publication: *SHELXTL* and *PLATON* (Spek, 2009 ▶).

## Supplementary Material

Crystal structure: contains datablock(s) global, I. DOI: 10.1107/S1600536813019776/lh5633sup1.cif

Structure factors: contains datablock(s) I. DOI: 10.1107/S1600536813019776/lh5633Isup2.hkl

Supplementary material file. DOI: 10.1107/S1600536813019776/lh5633Isup3.cml

Additional supplementary materials:  crystallographic information; 3D view; checkCIF report

## Figures and Tables

**Table 1:** Hydrogen-bond geometry (Å, °)

*D*—H⋯*A*	*D*—H	H⋯*A*	*D*⋯*A*	*D*—H⋯*A*
N4—H4*A*⋯O2	0.90 (2)	1.92 (2)	2.756 (3)	154 (3)
N1—H1⋯O1	0.90 (2)	1.78 (2)	2.550 (3)	141 (3)
N2—H2*B*⋯N5	1.02 (3)	1.84 (3)	2.858 (3)	174 (3)
O5—H5⋯O6	0.85 (2)	1.74 (2)	2.549 (3)	158 (4)
N5—H5*A*⋯O6	0.83 (2)	2.44 (3)	2.719 (3)	101 (2)
N5—H5*A*⋯O4^i^	0.83 (2)	2.46 (2)	3.162 (3)	143 (3)
N2—H2*A*⋯O1^ii^	0.94 (2)	2.08 (3)	2.762 (3)	128 (2)

Symmetry codes: (i) 
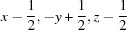
; (ii) 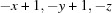.

**Fig. 1.**
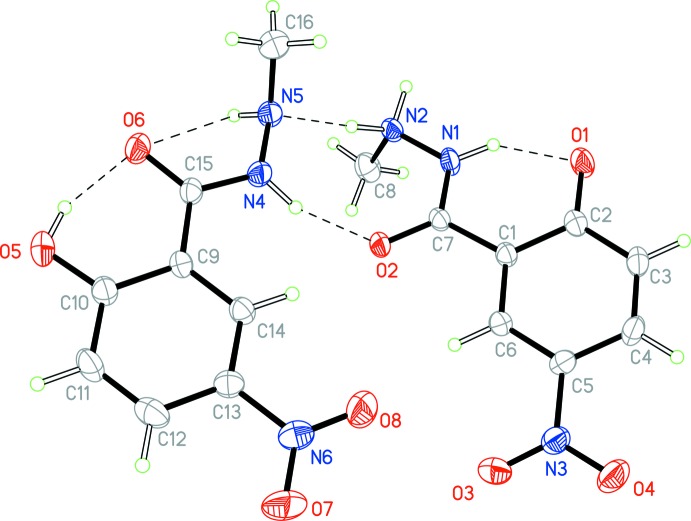
The asymmetric unit of (I) showing 30% probability displacement ellipsoids. Dashed lines indicate hydrogen bonds.

**Fig. 2.**
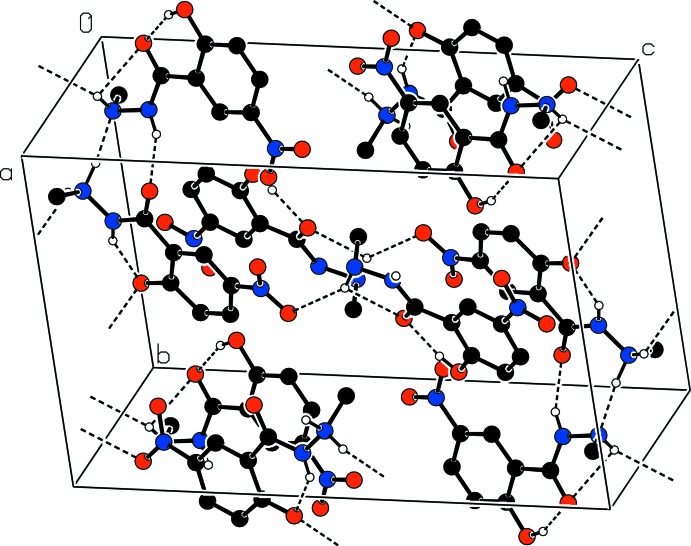
Part of the crystal structure with hydrogen bonds shown as dashed lines

## supplementary crystallographic information

### Comment

Derivatives of salicylhydrazide exhibit prominent biological activites such as antimicrobial activity, inhibitor of receptor and enzyme inhibitor (Bagchi *et al.*, 2004; Thompson *et al.*, 2004; Al-Mawsawi *et al.*, 2007). Several coordination sites exist in these compounds to lead to potential supramolecular structures. In our previous work, some anologs and their metal complexes were successfully sythesized and some latent functional studies were made (Jin *et al.*, 2006*a*,*b*). As part of our ongoing studies, the preparation and X-ray structure determination of the title compound (I) was undertaken.

The asymmetric unit of (I) is shown in Fig. 1. There are two molecules in the asymmetric unit. One of the molecules is in the zwitterionic form. In the zwitterion N2 is protonated and O1 deprotonated. The bond lengths and angles in each molecule are unexceptional and agree with those reported for similar structures (Liu *et al.*, 2006; Luo *et al.*, 2007; Xu & Liu, 2006; Zhang, 2012). In each molecule the non-H atoms, with the exception of the methyl and nitro groups, lie in an approximate plane with r.m.s deviations of 0.073 and 0.104 Å. The zwitterion contains an intramolecular N—H···O hydrogen bond and the other molecule contains intramolecular O—H···O and N—H···O hydrogen bonds. In the crystal, N—H···O and N—H···N hydrogen bonds link the molecules formimg a two-dimensional network (see Fig. 2) parallel to (101).

### Experimental

The title compound was systhesized by refluxing a mixture of ethyl 5-nitro-salicylate (0.1 mol) and methylhydrazine (0.2 mol) for 14 h. To the resulting solution acetic acid was added and it was cooled to room temperature. The mixture was evaporated and washed with diethyl ether. Single crystals suitable for X-ray diffraction analysis were grown by slow evaporation from a solution of (I) in methanol at room temperature.

### Refinement

H atoms bonded to C atoms were placed in calculated positions, with C—H distances of 0.93–0.96 Å. They were included in the refinement in the riding-model approximation, with isotropic displacement parameters set to 1.2*U*_eq_ of the carrier atom (1.5*U*_eq_ for CH_3_ H atoms). The amide and hydroxy H atoms were located in a difference Fourier map and refined isotropically.

### Crystal data

**Table:**

C_8_H_9_N_3_O_4_	*F*(000) = 880
*M**_r_* = 211.18	*D*_x_ = 1.556 Mg m^−^^3^
Monoclinic, *P*2_1_/*n*	Mo *K*α radiation, λ = 0.71073 Å
Hall symbol: -P 2yn	Cell parameters from 1664 reflections
*a* = 7.3818 (15) Å	θ = 2.9–21.8°
*b* = 13.106 (3) Å	µ = 0.13 mm^−^^1^
*c* = 18.719 (4) Å	*T* = 293 K
β = 95.25 (3)°	Block, yellow
*V* = 1803.4 (6) Å^3^	0.26 × 0.20 × 0.10 mm
*Z* = 8	

### Data collection

**Table:**

Bruker SMART CCD diffractometer	3536 independent reflections
Radiation source: fine-focus sealed tube	2356 reflections with *I* > 2σ(*I*)
Graphite monochromator	*R*_int_ = 0.044
φ and ω scans	θ_max_ = 26.0°, θ_min_ = 1.9°
Absorption correction: multi-scan (*SADABS*; Sheldrick, 1996)	*h* = −9→9
*T*_min_ = 0.968, *T*_max_ = 0.987	*k* = −15→16
11293 measured reflections	*l* = −23→16

### Refinement

**Table:**

Refinement on *F*^2^	Primary atom site location: structure-invariant direct methods
Least-squares matrix: full	Secondary atom site location: difference Fourier map
*R*[*F*^2^ > 2σ(*F*^2^)] = 0.058	Hydrogen site location: inferred from neighbouring sites
*wR*(*F*^2^) = 0.142	H atoms treated by a mixture of independent and constrained refinement
*S* = 1.06	*w* = 1/[σ^2^(*F*_o_^2^) + (0.0565*P*)^2^ + 0.5317*P*] where *P* = (*F*_o_^2^ + 2*F*_c_^2^)/3
3536 reflections	(Δ/σ)_max_ = 0.026
291 parameters	Δρ_max_ = 0.22 e Å^−^^3^
5 restraints	Δρ_min_ = −0.18 e Å^−^^3^

### Special details

**Table:**

Geometry. All e.s.d.'s (except the e.s.d. in the dihedral angle between two l.s. planes) are estimated using the full covariance matrix. The cell e.s.d.'s are taken into account individually in the estimation of e.s.d.'s in distances, angles and torsion angles; correlations between e.s.d.'s in cell parameters are only used when they are defined by crystal symmetry. An approximate (isotropic) treatment of cell e.s.d.'s is used for estimating e.s.d.'s involving l.s. planes.
Refinement. Refinement of *F*^2^ against ALL reflections. The weighted *R*-factor *wR* and goodness of fit *S* are based on *F*^2^, conventional *R*-factors *R* are based on *F*, with *F* set to zero for negative *F*^2^. The threshold expression of *F*^2^ > σ(*F*^2^) is used only for calculating *R*-factors(gt) *etc*. and is not relevant to the choice of reflections for refinement. *R*-factors based on *F*^2^ are statistically about twice as large as those based on *F*, and *R*-factors based on ALL data will be even larger.

### Fractional atomic coordinates and isotropic or equivalent isotropic displacement parameters (Å^2^)

**Table:**

	*x*	*y*	*z*	*U*_iso_*/*U*_eq_	
C1	0.5851 (3)	0.42908 (18)	0.18014 (13)	0.0339 (6)	
C2	0.5537 (4)	0.53339 (19)	0.15979 (15)	0.0422 (7)	
C3	0.5864 (4)	0.6078 (2)	0.21338 (15)	0.0472 (7)	
H3	0.5608	0.6757	0.2021	0.057*	
C4	0.6544 (4)	0.5839 (2)	0.28124 (15)	0.0445 (7)	
H4	0.6754	0.6347	0.3157	0.053*	
C5	0.6921 (3)	0.4822 (2)	0.29859 (14)	0.0377 (6)	
C6	0.6547 (3)	0.40610 (19)	0.24911 (13)	0.0358 (6)	
H6	0.6766	0.3385	0.2622	0.043*	
C7	0.5512 (3)	0.34258 (18)	0.12929 (13)	0.0336 (6)	
C8	0.6571 (4)	0.2470 (2)	−0.00925 (16)	0.0549 (8)	
H8A	0.7340	0.3000	−0.0249	0.082*	
H8B	0.6334	0.1979	−0.0471	0.082*	
H8C	0.7167	0.2139	0.0322	0.082*	
C9	0.3376 (3)	−0.01241 (18)	0.22768 (14)	0.0341 (6)	
C10	0.3287 (4)	−0.1136 (2)	0.25337 (15)	0.0427 (7)	
C11	0.3917 (4)	−0.1365 (2)	0.32380 (17)	0.0559 (8)	
H11	0.3873	−0.2034	0.3400	0.067*	
C12	0.4595 (4)	−0.0621 (2)	0.36920 (17)	0.0557 (8)	
H12	0.5013	−0.0778	0.4163	0.067*	
C13	0.4657 (4)	0.0376 (2)	0.34444 (14)	0.0421 (7)	
C14	0.4053 (3)	0.06248 (19)	0.27498 (14)	0.0382 (6)	
H14	0.4099	0.1299	0.2597	0.046*	
C15	0.2762 (3)	0.00751 (19)	0.15166 (14)	0.0363 (6)	
C16	0.0855 (4)	0.1430 (2)	0.03122 (17)	0.0601 (9)	
H16A	0.0122	0.0904	0.0497	0.090*	
H16B	0.0630	0.1455	−0.0201	0.090*	
H16C	0.0550	0.2076	0.0511	0.090*	
N1	0.5069 (3)	0.37013 (16)	0.06155 (12)	0.0432 (6)	
H1	0.489 (4)	0.4367 (15)	0.0517 (16)	0.072*	
N2	0.4833 (3)	0.29164 (17)	0.00913 (12)	0.0400 (5)	
H2A	0.420 (4)	0.321 (2)	−0.0313 (12)	0.072*	
H2B	0.409 (4)	0.233 (2)	0.0275 (16)	0.072*	
N3	0.7769 (3)	0.45572 (19)	0.36861 (12)	0.0463 (6)	
N4	0.3109 (3)	0.09987 (16)	0.12471 (12)	0.0422 (6)	
H4A	0.393 (3)	0.143 (2)	0.1470 (15)	0.072*	
N5	0.2755 (3)	0.12117 (16)	0.05067 (12)	0.0421 (6)	
H5A	0.306 (4)	0.0673 (17)	0.0315 (16)	0.072*	
N6	0.5389 (3)	0.1169 (2)	0.39330 (13)	0.0570 (7)	
O1	0.4975 (3)	0.55896 (14)	0.09395 (10)	0.0593 (6)	
O2	0.5657 (3)	0.25254 (12)	0.14945 (10)	0.0452 (5)	
O3	0.8350 (3)	0.36913 (18)	0.37959 (11)	0.0684 (7)	
O4	0.7919 (3)	0.52224 (16)	0.41560 (11)	0.0664 (6)	
O5	0.2604 (3)	−0.19058 (14)	0.21215 (12)	0.0598 (6)	
H5	0.223 (5)	−0.159 (3)	0.1736 (14)	0.090*	
O6	0.1955 (3)	−0.05861 (14)	0.11305 (10)	0.0507 (5)	
O7	0.5987 (4)	0.0933 (2)	0.45398 (13)	0.0933 (9)	
O8	0.5409 (4)	0.20419 (19)	0.37205 (13)	0.0902 (9)	

### Atomic displacement parameters (Å^2^)

**Table:**

	*U*^11^	*U*^22^	*U*^33^	*U*^12^	*U*^13^	*U*^23^
C1	0.0403 (14)	0.0276 (13)	0.0339 (15)	0.0005 (10)	0.0036 (11)	0.0025 (11)
C2	0.0498 (16)	0.0315 (15)	0.0446 (17)	0.0026 (12)	0.0010 (13)	0.0023 (13)
C3	0.0621 (19)	0.0282 (15)	0.0507 (19)	0.0000 (12)	0.0016 (15)	−0.0019 (13)
C4	0.0556 (17)	0.0333 (15)	0.0443 (17)	−0.0073 (12)	0.0033 (14)	−0.0107 (13)
C5	0.0383 (14)	0.0418 (15)	0.0327 (15)	−0.0069 (11)	0.0009 (11)	−0.0009 (12)
C6	0.0388 (14)	0.0310 (14)	0.0373 (15)	−0.0015 (11)	0.0013 (12)	0.0036 (12)
C7	0.0423 (15)	0.0271 (14)	0.0309 (15)	0.0015 (10)	0.0013 (11)	0.0042 (11)
C8	0.067 (2)	0.0474 (17)	0.0516 (19)	0.0106 (15)	0.0131 (16)	0.0002 (15)
C9	0.0372 (14)	0.0276 (13)	0.0379 (15)	0.0025 (10)	0.0056 (11)	0.0001 (12)
C10	0.0430 (15)	0.0366 (15)	0.0483 (18)	−0.0012 (12)	0.0033 (13)	0.0046 (13)
C11	0.064 (2)	0.0409 (17)	0.062 (2)	−0.0032 (14)	0.0016 (16)	0.0222 (16)
C12	0.0602 (19)	0.060 (2)	0.0470 (19)	0.0040 (15)	0.0020 (15)	0.0199 (16)
C13	0.0446 (16)	0.0467 (17)	0.0349 (16)	0.0002 (12)	0.0030 (12)	0.0007 (13)
C14	0.0461 (15)	0.0346 (14)	0.0341 (15)	0.0024 (11)	0.0046 (12)	0.0029 (12)
C15	0.0430 (15)	0.0285 (14)	0.0374 (16)	0.0007 (11)	0.0030 (12)	−0.0033 (12)
C16	0.067 (2)	0.057 (2)	0.054 (2)	−0.0022 (16)	−0.0081 (16)	−0.0028 (16)
N1	0.0739 (16)	0.0235 (11)	0.0315 (13)	0.0052 (11)	0.0010 (11)	0.0010 (10)
N2	0.0568 (15)	0.0314 (12)	0.0304 (13)	0.0039 (10)	−0.0032 (11)	0.0032 (10)
N3	0.0541 (15)	0.0462 (15)	0.0379 (15)	−0.0087 (12)	0.0004 (11)	−0.0008 (12)
N4	0.0580 (15)	0.0364 (13)	0.0305 (13)	−0.0075 (10)	−0.0048 (11)	0.0004 (10)
N5	0.0590 (15)	0.0362 (13)	0.0297 (13)	−0.0020 (11)	−0.0023 (11)	−0.0008 (10)
N6	0.0685 (17)	0.0639 (19)	0.0370 (16)	−0.0011 (14)	−0.0036 (13)	−0.0037 (14)
O1	0.1027 (17)	0.0323 (10)	0.0399 (12)	0.0062 (10)	−0.0102 (11)	0.0077 (9)
O2	0.0681 (13)	0.0256 (10)	0.0391 (11)	−0.0029 (8)	−0.0099 (9)	0.0051 (8)
O3	0.0945 (17)	0.0608 (15)	0.0451 (13)	0.0100 (13)	−0.0185 (12)	0.0041 (11)
O4	0.0981 (17)	0.0579 (14)	0.0412 (13)	−0.0164 (12)	−0.0037 (12)	−0.0104 (11)
O5	0.0765 (15)	0.0308 (11)	0.0702 (16)	−0.0083 (10)	−0.0033 (12)	0.0015 (11)
O6	0.0681 (13)	0.0373 (11)	0.0454 (12)	−0.0088 (9)	−0.0029 (10)	−0.0066 (9)
O7	0.131 (2)	0.102 (2)	0.0402 (14)	−0.0151 (17)	−0.0271 (14)	0.0047 (14)
O8	0.155 (3)	0.0508 (15)	0.0583 (16)	−0.0083 (15)	−0.0239 (16)	−0.0097 (13)

### Geometric parameters (Å, º)

**Table:**

C1—C6	1.379 (3)	C11—H11	0.9300
C1—C2	1.432 (3)	C12—C13	1.389 (4)
C1—C7	1.487 (3)	C12—H12	0.9300
C2—O1	1.308 (3)	C13—C14	1.375 (4)
C2—C3	1.404 (4)	C13—N6	1.455 (4)
C3—C4	1.359 (4)	C14—H14	0.9300
C3—H3	0.9300	C15—O6	1.245 (3)
C4—C5	1.394 (4)	C15—N4	1.345 (3)
C4—H4	0.9300	C16—N5	1.445 (4)
C5—C6	1.372 (3)	C16—H16A	0.9600
C5—N3	1.443 (3)	C16—H16B	0.9600
C6—H6	0.9300	C16—H16C	0.9600
C7—O2	1.240 (3)	N1—N2	1.421 (3)
C7—N1	1.330 (3)	N1—H1	0.899 (17)
C8—N2	1.479 (3)	N2—H2A	0.937 (17)
C8—H8A	0.9600	N2—H2B	1.02 (3)
C8—H8B	0.9600	N3—O3	1.224 (3)
C8—H8C	0.9600	N3—O4	1.236 (3)
C9—C14	1.384 (3)	N4—N5	1.415 (3)
C9—C10	1.414 (3)	N4—H4A	0.903 (18)
C9—C15	1.477 (3)	N5—H5A	0.832 (17)
C10—O5	1.340 (3)	N6—O8	1.212 (3)
C10—C11	1.390 (4)	N6—O7	1.220 (3)
C11—C12	1.359 (4)	O5—H5	0.854 (18)
			
C6—C1—C2	119.7 (2)	C13—C12—H12	120.4
C6—C1—C7	117.3 (2)	C14—C13—C12	121.4 (3)
C2—C1—C7	123.0 (2)	C14—C13—N6	119.7 (2)
O1—C2—C3	121.0 (2)	C12—C13—N6	118.9 (3)
O1—C2—C1	121.7 (2)	C13—C14—C9	120.3 (2)
C3—C2—C1	117.4 (2)	C13—C14—H14	119.9
C4—C3—C2	122.2 (3)	C9—C14—H14	119.9
C4—C3—H3	118.9	O6—C15—N4	120.4 (2)
C2—C3—H3	118.9	O6—C15—C9	121.7 (2)
C3—C4—C5	119.2 (2)	N4—C15—C9	117.9 (2)
C3—C4—H4	120.4	N5—C16—H16A	109.5
C5—C4—H4	120.4	N5—C16—H16B	109.5
C6—C5—C4	121.0 (2)	H16A—C16—H16B	109.5
C6—C5—N3	118.8 (2)	N5—C16—H16C	109.5
C4—C5—N3	120.2 (2)	H16A—C16—H16C	109.5
C5—C6—C1	120.5 (2)	H16B—C16—H16C	109.5
C5—C6—H6	119.7	C7—N1—N2	117.7 (2)
C1—C6—H6	119.7	C7—N1—H1	119 (2)
O2—C7—N1	123.7 (2)	N2—N1—H1	124 (2)
O2—C7—C1	121.7 (2)	N1—N2—C8	113.2 (2)
N1—C7—C1	114.6 (2)	N1—N2—H2A	106.1 (19)
N2—C8—H8A	109.5	C8—N2—H2A	110.8 (19)
N2—C8—H8B	109.5	N1—N2—H2B	110.2 (17)
H8A—C8—H8B	109.5	C8—N2—H2B	106.7 (17)
N2—C8—H8C	109.5	H2A—N2—H2B	110 (3)
H8A—C8—H8C	109.5	O3—N3—O4	121.8 (2)
H8B—C8—H8C	109.5	O3—N3—C5	119.5 (2)
C14—C9—C10	118.2 (2)	O4—N3—C5	118.7 (2)
C14—C9—C15	123.5 (2)	C15—N4—N5	121.4 (2)
C10—C9—C15	118.3 (2)	C15—N4—H4A	122 (2)
O5—C10—C11	117.3 (2)	N5—N4—H4A	113 (2)
O5—C10—C9	122.5 (2)	N4—N5—C16	111.9 (2)
C11—C10—C9	120.2 (3)	N4—N5—H5A	103 (2)
C12—C11—C10	120.7 (3)	C16—N5—H5A	111 (2)
C12—C11—H11	119.6	O8—N6—O7	122.1 (3)
C10—C11—H11	119.6	O8—N6—C13	118.9 (2)
C11—C12—C13	119.2 (3)	O7—N6—C13	119.1 (3)
C11—C12—H12	120.4	C10—O5—H5	102 (3)
			
C6—C1—C2—O1	176.1 (2)	C11—C12—C13—C14	−0.2 (4)
C7—C1—C2—O1	−1.9 (4)	C11—C12—C13—N6	−179.9 (3)
C6—C1—C2—C3	−3.7 (4)	C12—C13—C14—C9	−0.5 (4)
C7—C1—C2—C3	178.3 (2)	N6—C13—C14—C9	179.1 (2)
O1—C2—C3—C4	−176.3 (3)	C10—C9—C14—C13	1.5 (4)
C1—C2—C3—C4	3.5 (4)	C15—C9—C14—C13	−177.8 (2)
C2—C3—C4—C5	−0.4 (4)	C14—C9—C15—O6	−170.9 (2)
C3—C4—C5—C6	−2.7 (4)	C10—C9—C15—O6	9.8 (4)
C3—C4—C5—N3	175.3 (2)	C14—C9—C15—N4	9.4 (4)
C4—C5—C6—C1	2.4 (4)	C10—C9—C15—N4	−169.9 (2)
N3—C5—C6—C1	−175.6 (2)	O2—C7—N1—N2	−3.0 (4)
C2—C1—C6—C5	0.8 (4)	C1—C7—N1—N2	176.3 (2)
C7—C1—C6—C5	179.0 (2)	C7—N1—N2—C8	−75.0 (3)
C6—C1—C7—O2	7.7 (4)	C6—C5—N3—O3	9.4 (4)
C2—C1—C7—O2	−174.2 (2)	C4—C5—N3—O3	−168.7 (3)
C6—C1—C7—N1	−171.6 (2)	C6—C5—N3—O4	−172.0 (2)
C2—C1—C7—N1	6.5 (3)	C4—C5—N3—O4	10.0 (4)
C14—C9—C10—O5	178.0 (2)	O6—C15—N4—N5	−7.0 (4)
C15—C9—C10—O5	−2.7 (4)	C9—C15—N4—N5	172.7 (2)
C14—C9—C10—C11	−1.8 (4)	C15—N4—N5—C16	80.1 (3)
C15—C9—C10—C11	177.5 (2)	C14—C13—N6—O8	1.8 (4)
O5—C10—C11—C12	−178.7 (3)	C12—C13—N6—O8	−178.5 (3)
C9—C10—C11—C12	1.1 (4)	C14—C13—N6—O7	−177.2 (3)
C10—C11—C12—C13	−0.1 (4)	C12—C13—N6—O7	2.5 (4)

### Hydrogen-bond geometry (Å, º)

**Table:**

*D*—H···*A*	*D*—H	H···*A*	*D*···*A*	*D*—H···*A*
N4—H4*A*···O2	0.90 (2)	1.92 (2)	2.756 (3)	154 (3)
N1—H1···O1	0.90 (2)	1.78 (2)	2.550 (3)	141 (3)
N2—H2*B*···N5	1.02 (3)	1.84 (3)	2.858 (3)	174 (3)
O5—H5···O6	0.85 (2)	1.74 (2)	2.549 (3)	158 (4)
N5—H5*A*···O6	0.83 (2)	2.44 (3)	2.719 (3)	101 (2)
N5—H5*A*···O4^i^	0.83 (2)	2.46 (2)	3.162 (3)	143 (3)
N2—H2*A*···O7^i^	0.94 (2)	2.61 (3)	3.296 (3)	130 (2)
N2—H2*A*···O1^ii^	0.94 (2)	2.08 (3)	2.762 (3)	128 (2)

Symmetry codes: (i) *x*−1/2, −*y*+1/2, *z*−1/2; (ii) −*x*+1, −*y*+1, −*z*.
